# Perception of maximum distance jumpable remains accurate after an intense physical exercise and during recovery

**DOI:** 10.3758/s13414-021-02315-z

**Published:** 2021-09-14

**Authors:** Jorn J. Flach, Anoek K. Schotborgh, Rob Withagen, Joanne Smith

**Affiliations:** Department of Human Movement Sciences, University Medical Center Groningen, University of Groningen, P.O. Box 196, 9700 AD Groningen, The Netherlands

**Keywords:** Affordances, Embodied perception, Fatigue, Action capabilities, Perception and action

## Abstract

Earlier studies have revealed that changes in action capabilities due to fatigue or wearing a backpack have an effect on the perception of distance in meters or steepness in angles. Although these findings are interesting by themselves, they leave us uninformed about whether the accuracy of affordance perception is affected by fatigue. Are people still capable of accurately perceiving the maximum distance jumpable after an intense physical exercise? In the present experiment, this question is addressed. We found that after maximal exertion in a squatting task, the actual maximum jumping distance significantly decreased, but recovered quickly. Interestingly, on average, the participants accurately perceived their maximum jumping distance both before and after the squatting task. Apparently, the accuracy of the affordance perception remains intact after an intense physical exercise. The implications of this finding are discussed.

## Introduction

In the 1960s, the ecological psychologist Gibson (1966, 1979/1986) asserted that animals perceive their environments in terms of affordances. Affordances refer to the action possibilities that the environment provides a certain animal. For example, for a human being, a chair affords sitting upon and a cup affords grasping. Importantly, and as emphasized by Gibson, affordances exist by virtue of the relationship between the action capabilities of the actor and the physical characteristics of the environment. Whether a chair affords sitting depends on the length and flexibility of the legs in relation to the height of the seat (e.g., Mark, 1987).

Since the introduction of the concept, multiple studies have examined whether humans are capable of accurately perceiving affordances. These studies have shown that participants are capable of perceiving the maximum stair-riser height they can climb (e.g., Mark, 1987; Warren, 1984), the maximum distance they can reach (e.g., Carello et al., 1989; Cole et al., 2013; Heft, 1993), the minimal width of the aperture they can pass through (Warren & Whang, 1987) and the maximum distance they can step (Chemero et al., 2003; Cole et al., 2013; Day et al., 2015; Wagman et al., 2016) and jump (Cole et al., 2013; Day et al., 2015; Wagman et al., 2016). Yet sometimes participants underestimated or overestimated their action capabilities in judgment tasks (e.g., Carello et al., 1989; Cole et al., 2013, but see Heft, 1993; Wagman et al., 2016).

Interestingly, action capabilities are dynamic and change at several different timescales. At the timescale of development, for example, action capabilities come and go (e.g., Adolph & Hoch, 2019; Goldfield, 1995; Thelen & Smith, 1994)—a baby learns to crawl and later to walk, and action capabilities tend to decline in elderly. However, also on a shorter timescale, there can be a *temporary* decline in action capabilities. For example, after an intensive physical exercise, the maximum power that the legs can produce is significantly reduced (see Skurvydas et al., 2008) which is likely to affect several action capabilities for a certain period of time.

Earlier studies have already shown that fatigue, induced by physical exercise, can have an effect on perception. More generally, in a series of experiments, Proffitt and colleagues have demonstrated that factors that affect the action capabilities of a participant (like fatigue and wearing a heavy backpack) can influence the perception of, among other things, steepness and distance (Bhalla & Proffitt, 1999; Proffitt, 2013; Proffitt et al., 2003). Bhalla and Proffitt (1999), for example, found that participants perceive a hill to be steeper after an exhausting run (but see Durgin et al., 2009, for an interesting critique on the studies of Profitt and colleagues). Although these studies provide interesting insights into perception, one might wonder whether the questions posed are that relevant in the context of understanding our daily behaviour. Indeed, and as emphasized by Gibson (1979/1986), to behave adaptively, animals have to perceive their environment not in terms of metric units but in terms of what they can do in it. And the studies by Proffitt and colleagues do not inform us about how the perception of those affordances is affected by changes in action capabilities. Are we capable of accurately perceiving our opportunities for action when we are fatigued?

To our knowledge, only the study by Pijpers et al. (2007) addressed the question of whether fatigue affects the perception of affordances. In a climbing task, they examined the perceived and actual maximum reaching height of participants. Interestingly, Pijpers et al. demonstrated that in the situation of participants climbing to exhaustion, the perceived reaching height largely followed the changes in the actual reaching height—when the actual reaching height declined due to the performed actions, the perceived reaching height declined as well. As a result, the participants were still capable of perceiving this affordance in a fatigued state.

In the present experiment, we examined the effects of fatigue in the paradigm of jumping (Cole et al., 2013; Wagman et al., 2016). Are participants capable of accurately perceiving their maximum jumping distance after a short, but intense physical exercise? In addition, we investigated whether and, if so, how the perception changes when the participants recover from this exercise. To that end, we asked participants to estimate their maximum jumping distance before and after having performed a squat for as long as possible. Comparing these estimations with their actual maximum jumping distance before and after the squat allows us to determine whether affordances are accurately perceived, also in the case of a temporary decline in jumping capacity. Because affordance perception is generally rather accurate, we, following Pijpers et al. (2007), hypothesized that also after an intense physical exercise, participants are still capable of accurately perceiving the maximum distance they can jump.

## Method

### Participants

Forty-one participants (24 men, 17 women; age *M* = 22.8 years, *SD* = 3.8, range: 18–38 years) volunteered to participate in this study. Effect sizes from Pijpers et al. (2007; Experiment 2) on the effects of fatigue on perception of affordances for reaching distance were partial eta squared of 0.39 and 0.24 for perceived reaching distance and actual reaching distance, respectively, supporting the expectation for large effects (under guidelines from Cohen, 1988). A power analysis using G*Power 3 (Faul et al., 2007) indicated that a minimum sample of eight to 13 participants would be needed to detect similar effects with 90% power using a one-way repeated-measures analysis of variance (ANOVA; six repeated measures of time) with alpha at .05.

Participants were free of injuries and wore comfortably fitting clothes to ensure full range of motion. The study protocol was approved by the local institute’s ethics committee. All participants gave informed consent.

### Materials and apparatus

The estimations of jumping distance and the actual maximum jumps were performed in an open space approximately 387 cm long. The floor surface was linoleum with no tiles, obvious joins or texture that participants could have used as a reference when making their judgments. The participant’s starting position was marked by a piece of tape on the floor. To determine estimated jumping distance, a cart with a small wooden plank attached was moved towards or away from the participant. The distance of estimations and actual jumps were measured using a measuring tape. A cycle-ergometer set at 50 watts was used for participants to warm up. Perceived exertion was measured using the Borg Rating of Perceived Exertion (RPE) scale, where participants are asked to rate their level of physical exertion on a scale ranging from 6 to 20, with 6 indicating *no exertion* and 20 *maximum exertion* (Borg, 1998). A stopwatch was used to measure time spent in the squat position and elapsed time between the end of the squat position and the start of each posttest trial.

### Design

A within-participant pretest versus posttest design was used to compare performance before and after maximal exertion in a squatting task. We opted for a squatting task because it is a high intensity activity involving the same leg muscles used in the jumping action, and rapid recovery is known to occur after a short, but maximally tiring squat exercise. Skurvydas et al. (2008) showed that directly after a maximum 1-minute isometric contraction of the quadriceps femoris with a knee angle of 90 degrees the voluntary maximum force was only 38% of the actual maximum power. After 3 minutes of rest, however, there was no longer any difference in the maximum force generated in relation to before the intervention. Therefore, it was expected that use of a squatting intervention in the present study would induce quick fatigue and rapid recovery of the leg muscles involved in the jumping action.

**Fig. 1:**
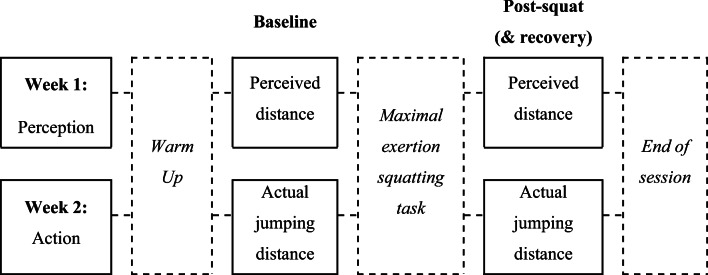
Diagram of experimental design

Perceived jumping distance and actual jumping distance were measured during two separate sessions one week apart (see Fig. 1). No jumps were performed on the first day of testing—after all, that would inform the participants about their actual jumping capabilities which is likely to immediately correct their judgments of this capacity if needed (see Cole et al., 2013; Day et al., 2015).

### Procedure

Before testing began, participants were first informed of the research question of the study—can people perceive the limit of their jumping distance if they cannot perform as well as usual. Participants were asked to judge the maximum distance they could jump in different conditions, they were asked to be as accurate as possible. A brief demonstration of the jumping action was performed submaximally by one of the experimenters, in order to demonstrate the type of jump to be performed—namely, with a two-footed take-off and landing. Participants were allowed to take one step at landing to keep balance, as long as they kept one foot at the landing location. Next, participants were instructed on how to answer the Borgscale.

#### Warm-up

To make sure that the participants would jump as far as they could (and to minimize risk of injury), we included a little warm-up to bring the action system into a proper state. The warm-up was identical on both days of measurements, to ensure all participants had a similar amount of exertion before testing started. Participants started each session by answering a Borgscale to measure baseline exertion levels. Next, they completed a short 5-minute warm-up on the cycle ergometer cycling at 75 rpm. Afterwards participants answered the Borgscale for a second time, to test whether the warm-up had the desired effect.

#### Baseline

Perceived and actual jumping distances were measured. In all trials, participants started *standing* behind the line on the floor, with their toes touching the line.

#### Perceived jumping distance (Week 1)

Within each trial, participants’ perceived jumping distance was measured using two consecutive estimations; in one estimate the experimenter moved the cart away from the participant, and in the other estimate the experimenter moved the cart towards the participant. Starting points for moving the cart were either 0 cm or 360 cm from the participant’s starting position. During each estimation, when the plank mounted to the cart reached the perceived jumping distance of a participant, they were instructed to say ‘stop’. A small correction could then be made by instructing the experimenter to move the cart a bit closer or a bit further away. After utilizing this possibility or not, participants were instructed to close their eyes. The distance between the tape on the floor and the plank on the cart was measured. For every trial, perceived jumping distance was then calculated as an average of these two estimates, in order to counteract ‘error of anticipation’ found in earlier research (see Chemero et al., 2003). This process was repeated three times; therefore, each participant completed three baseline perceived jumping distance trials.

#### Actual maximal jumping distance (Week 2)

Participants performed three maximum jumps at their own pace to determine their baseline actual maximal jumping distance. Jumping distance was measured as the distance between the starting line and either the heel of the foot closest to the starting line at landing, or, if a step was required, the heel of the foot that was still in the original landing location would be measured as jumped distance. After each jump was measured participants would return to the starting line.

#### Maximal exertion squat

The maximal exertion squat was identical on both days of measurements. Participants entered a squat position with knees at a 90-degree angle. They were instructed to hold this position for as long as possible, while being encouraged by the experimenters to keep going. Time spent in this position was measured. When participants could no longer hold this position, they would answer the Borgscale for a third time.

#### Posttest

Perceived and actual jumping distances were measured directly after the maximal exertion squat, using the same method as the baseline measurements. Moreover, to ensure that participants had similar amounts of recuperation time on both days, (i) in Week 1 (perceived jumping distance), we measured the time between the end of the squat and each of the estimates used for the perceived jumping distance trials; this was recorded as the ‘*time post-squat*’ for that perceived jumping distance trial. Then (ii), in Week 2 (actual maximal jumping distance), participants were asked to jump at the ‘*time post-squat*’ that corresponded to each of their Week 1 perceived jumping distance trials.

#### End of testing session

At the end of both measurement days, participants answered the Borgscale for the fourth time to check for recovery.

### Data analysis

Analysis was performed using SPSS Version 19.0. Participants were excluded from analysis if they (1) scored lower than 15 on the post-squat Borgscale in either the perception or action trials (this was to ensure they had put in the requested effort during the squat); (2) did not correctly perform the required jump; (3) jumped at the wrong time post-intervention; or (4) stepped away at landing to the point that determining the covered distance of the jump was no longer possible.

To confirm that (i) perceived exertion did not differ between both days of measurements and (ii) the squat intervention did indeed induce fatigue, all Borgscale scores were compared using a 2 × 4 repeated-measures ANOVA, with session (perception, action) and time (pre-warm-up, post-warm-up, post-squat, and end of session) as within-subjects factors.

To investigate whether the squat was an effective intervention for reducing actual jumping distance, and whether it had an effect on perceived jumping distance, three dependent variables were analysed: *actual jumping distance*, *perceived jumping distance*, and the relative *ratio of perceived and actual jumping distance* (calculated by dividing a perceived jumping distance by its corresponding actual jumping distance). Each dependent variable was subjected to one-way repeated-measures ANOVA, with time (Baseline1, Baseline2, Baseline3, Post-squat1, Post-squat2, and Post-squat3) as within-subjects factors. Repeated contrasts were used to focus on the following comparisons: (i) establish *baseline* performance (Baseline1 vs. Baseline2, and Baseline2 vs. Baseline3); (ii) determine *direct effects of squat* (Baseline3 vs. Post-squat1); and (iii) establish extent of *recovery* (Post-squat1 vs. Post-squat2, and Post-squat2 vs. Post-squat3). Finally, to test whether the relative ratio of perceived and actual jumping distance was accurate, one-sample *t* tests were used to compare all ratios to a value of 1, which would be a perfect estimation.

The significance level for all statistical tests was set at α < .05. With regard to the ANOVAs, if the assumption of sphericity was violated the Greenhouse–Geisser correction was applied. Effect sizes were calculated using partial eta-squared, η_p_^2^, and generalized eta-squared, η_G_^2^ (Bakeman, 2005; Lakens, 2013), and were interpreted according to Cohen’s recommendation of 0.02 for a small effect, 0.13 for a medium effect, and 0.26 for a large effect (Cohen, 1988). For the t-test measures of effect size (r) were used. We used Bonferroni correction when multiple post-hoc t-tests were performed.

## Results

Five participants were excluded from the data analysis based on the exclusion criteria described in the Methods section; Criteria 1 (*n* = 2), Criteria 2–4 (*n* = 1 for each). In total, 36 participants (22 men, 14 women; age *M* = 23.1 years, *SD* = 3.9 years) were included in the final data analysis.

### Perceived exertion

**Fig. 2:**
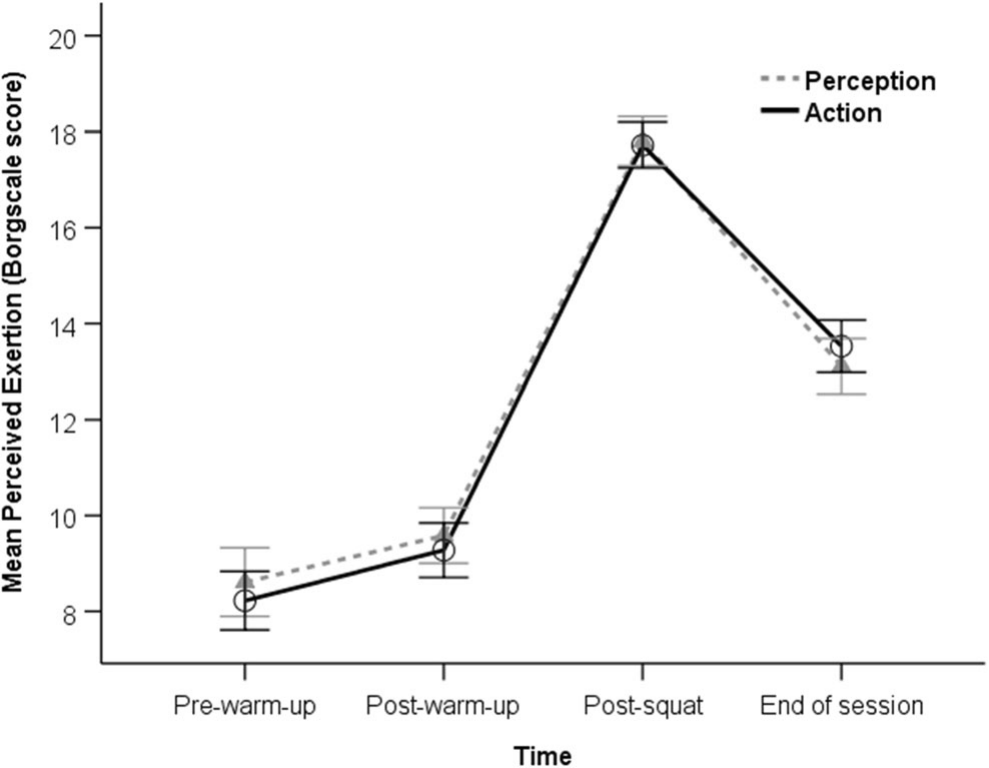
Mean Borgscale scores (*N* = 36) indicating levels of perceived physical exertion reported during the perception and action testing sessions. The scale ranges from 6 (indicating *no exertion*) to 20 (*maximum exertion)*. Error bars indicate 95% confidence intervals

Repeated measures ANOVA showed no main effect of session on Borgscale score, *F*(1, 35) = 0.15, *p* = .704, and no Session × Time interaction, *F*(2.16, 75.48) = 2.73, *p* = .068. This confirms that participant’s perceived exertion did not differ between perception and action sessions (see Fig. 2). As expected, there was a significant main effect of time, *F*(2.01, 70.27) = 344.84, *p* < .001, η_p_^2^ = .91, η_G_^2^ = .86. Repeated contrasts on perceived exertion confirmed: from pre-warm-up to post-warm-up, a significant increase, *F*(1, 35) = 33.01, *p* < .001, η_p_^2^ = .49; from post-warm-up to post-squat, a large significant increase, *F*(1, 35) = 546.88, *p* < .001, η_p_^2^ = .94; and from post-squat to the end of session, a significant decrease, *F*(1, 35) = 209.35, *p* < .001, η_p_^2^ = .86. Together, these results confirm the squatting task significantly increased the participants perceived exertion, which then decreased again before the end of the testing session.

### Actual jumping distance

**Fig. 3:**
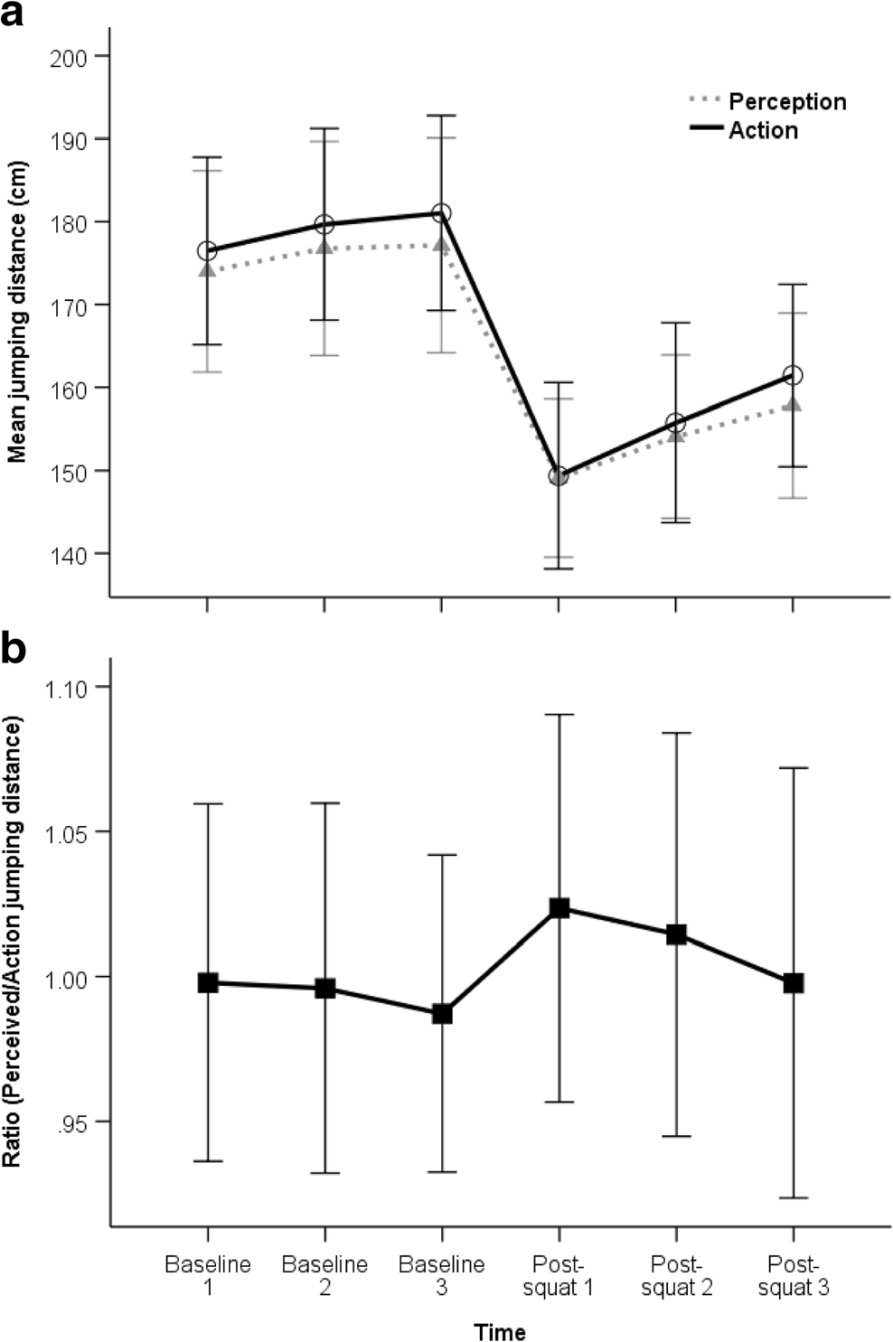
Mean perceived and actual jumping distances (**a**), and ratio of perceived/action jumping distance (**b**), for baseline, post-squat, and recovery measurements. Bars indicate 95% confidence intervals

Figure 3a shows the mean distances jumped at baseline, post-squat intervention, and during recovery. A repeated-measures ANOVA showed a significant main effect of time on actual jumping distance, *F*(2.45, 85.59) = 43.80, *p* < .001, η_p_^2^ = .56, η_G_^2^ = .35. Results of the repeated contrasts are reported separately for each phase of the testing.

#### Baseline

No significant differences in jumping distance were found between the three jumps pre-intervention—namely, between Baseline1 and Baseline2, *F*(1, 35) = 3.03, *p* = .090, and between Baseline2 and Baseline3, *F*(1, 35) = 0.96, *p* = .334. This confirms participants exhibited a stable baseline jumping performance.

#### Effect of squat

The maximal jumping distance was significantly shorter, *F*(1, 35) = 73.56, *p* <.001, η_p_^2^ = .68, immediately after the squat (Post-squat1: *M* = 149.4, *SD* = 33.2; range: 82–221 cm) compared with before the squat (Baseline3: *M* = 181.0, *SD* = 34.7; range: 106–252 cm). Therefore, the squat had the desired effect of significantly reducing jumping capacity, by approximately 32 cm.

#### Recovery

Significant differences in jumping distance were found between the three jumps post-intervention—namely, Post-squat2 was larger than Post-squat1, *F*(1, 35) = 6.95, *p* = .012, η_p_^2^ = .17, and Post-squat3 was larger than Post-squat2, *F*(1, 35) = 7.35, *p* = .010, η_p_^2^ = .17. In addition, Bonferroni post hoc *t* tests showed that Post-squat3 was larger than Post-squat1, and moreover, Post-squat1–3 were all smaller than Baseline1–3 (all corrected *p* values ≤ .001). This confirms that recovery did occur post-intervention, with actual distances jumped increasing over time (by approximately 12 cm); however, they did not fully recover to pre-squat/baseline levels (instead they were approximately 20 cm shorter than pre-squat/baseline).

### Perceived distance

Figure 3a shows the mean perceived distances at baseline, post-squat intervention, and during recovery. A repeated-measures ANOVA showed a significant main effect of time on perceived distance, *F*(1.94, 67.79) = 35.20, *p* <.001, η_p_^2^ = .50, η_G_^2^ = .31. The repeated contrasts are reported separately for each phase of the testing.

#### Baseline

No significant differences in perceived distance were found between the three trials pre-intervention—namely, between Baseline1 and Baseline2, *F*(1, 35) = 2.59, *p* =.117, and between Baseline2 and Baseline3, *F*(1, 35) = 0.05, *p* =.821. This confirms participants exhibited a stable baseline perceived distance.

#### Effect of squat

The perceived distance was significantly shorter, *F*(1, 35) = 56.89, *p* < .001, η_p_^2^ = .62, immediately after the squat (Post-squat1: *M* = 149.1, *SD* = 28.2; range: 103.5–212 cm) compared with before the squat (Baseline3: *M* = 177.1, *SD* = 38.3; range: 106–250 cm). Therefore, the squat had the expected effect of significantly lowering perceived jumping distance, by approximately 28 cm.

#### Recovery

Significant differences in perceived jumping distance were found between the three jumps post-intervention–namely, Post-squat2 was larger than Post-squat1, *F*(1, 35) = 7.60, *p* = .009, η_p_^2^ = .18, and Post-squat3 was larger than Post-squat2, *F*(1, 35) = 4.68, *p* = .038, η_p_^2^ = .12. In addition, Bonferroni post hoc *t* tests showed Post-squat3 was larger than Post-squat1 (*p* = .011), moreover Post-squat1–3 were all smaller than Baseline1–3 (*p*s < .001). This indicates that recovery did occur post-intervention, with perceived jumping distance increasing over time (by approximately 9 cm); however, it did not recover to pre-squat/baseline levels (instead, they were approximately 19 cm shorter than pre-squat/baseline).^1^

### Ratio of perceived and actual jumping distance

Figure 3b shows the mean ratio of perceived to actual jumping distance for baseline and post-squat trials. A repeated-measures ANOVA showed no main effect of time on ratio, *F*(2.73, 95.51) = 0.56, *p* = .627. One-sample *t* tests comparing all ratios to 1 were also nonsignificant (*p* values > .05), meaning no relative differences were found between perceived distance and actual jumping distance. Apparently, participants seem to adjust their perceived maximum jumping distance to the same degree as their change in actual jumping distance.

**Fig. 4:**
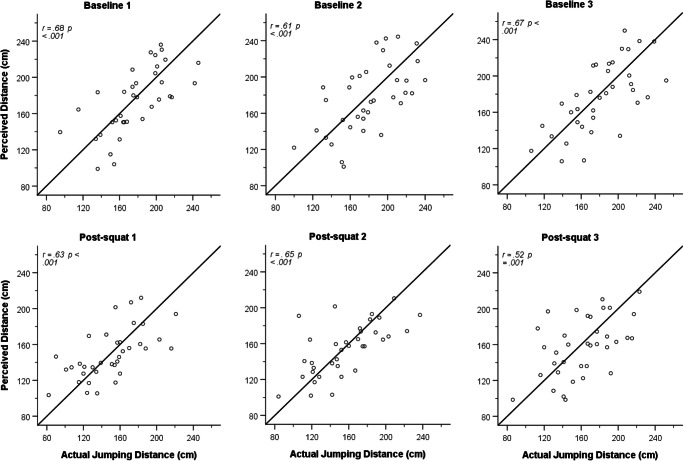
Scatter plots of participants’ perceived and actual maximum jumping distances at baseline (top row) and post-squat (bottom row). The diagonal line represents prefect agreement between perceived and actual jumping distance

This effect is also observed when computing the Pearson correlations between perceived and actual jumping distance for each of the baseline and post-squat intervention trials. Significant correlations between perceived and actual jumping distance for baseline (*r*s = .61 to .68, *p*s ≤ .001) and post-squat (*r*s = .52 to .65, *p*s ≤ .001) showed large effect sizes (see Fig. 4 for scatter plots showing participants’ perceived and actual jumping distance at baseline and post-squat).

## Discussion

In the present study, we tested whether the perception of the maximum distance jumpable remains accurate after an intense physical exercise. To that end, participants were to judge their maximum distance jumpable before and after holding a squat position for as long as possible. That physical intervention resulted in a temporary decline of the jumping capabilities—immediately after the intervention, the actual jumping distance significantly decreased, but recovered during the posttest of the experiment. Interestingly, we found that, on average, the perceived distance jumpable closely followed the actual distance jumpable, also in the recovery phase. In the remainder of the discussion, we will explore the implications of these findings.

### The adaptiveness of affordance perception

Proffitt and colleagues have conducted several studies that examined the effects of changes in the action capabilities on perception (e.g., Bhalla & Proffitt, 1999; Proffitt et al., 2003). They have asked participants to report the distance in meters or the steepness in angles, and demonstrated these judgements are affected by (temporary) changes of the participants’ action capabilities. However, and as mentioned in the Introduction, although interesting, one might wonder how relevant these effects are for the behaving animal. After all, and as already implied by Gibson’s (1966, 1979/1986) evolutionarily inspired ecological approach, to survive and reproduce, perceiving the environment in metric units like meters and angles is not that relevant. Rather, what the agent has to perceive is what she can do in the environment. That is, from an evolutionary perspective, affordances are likely to be the primary objects of perception (see, e.g., Reed, 1996; Withagen & Chemero, 2009).

Hence, our finding that on average the perception of the maximum distance jumpable is still accurate, both immediately after the squat and during the recovery phase, is arguably even more interesting than the earlier reported effects. Together with Pijpers et al.’s (2007) study which showed that the perceived distance reachable follows changes in actual distance reachable, it suggests that the perception of affordances is still adaptive even if an agent is fatigued. Hence, although the accuracy of the perceptual judgments of distances in terms of meters (Proffitt et al., 2003) or steepness in terms of angles (Bhalla & Proffitt, 1999) is affected by changes in the action capabilities, the perception of affordances appears to adaptively follow, and remain accurate relative to, these changes in action capabilities.

But to what extent can we be certain that the participants’ affordance perception was adequately adjusted to the decline in jumping capacities? Is it not also possible that the participants’ perception was unaltered but that they simply adjusted their responses? Indeed, the critique that has been leveled against Proffitt and colleagues could equally be applied to the present study. As touched upon in the introduction, Durgin et al. (2009) showed that wearing a backpack mainly affected the slope perception if the participants believed that the purpose of the study was to examine the effects of the backpack on the perception of slope. If the participant was persuaded that the purpose of the backpack was to carry the measurement equipment for monitoring their muscle activity, then the perception of slope was unaffected. Hence, Durgin et al. surmised that the effects that were reported by Proffitt and colleagues might have been the result of “the social, not physical, demands of the experimental context” (p. 964; but see Proffitt, 2009, for an interesting critique). In like fashion, the participants in our study were informed that we were interested in whether their perception of the maximum distance jumpable was still accurate after an intense physical exercise. Hence, it might be that the participants’ perception of the maximum distance jumpable was unaltered by the squatting, yet the participants simply judged the maximum distance to be a bit shorter (and continually increased it when the posttest progressed) to behave in keeping with the expectations of the experimenters. Although we cannot exclude this possibility based on the present experiment, our observation that, on average, the maximum distance jumpable is judged so accurately over the course of the entire experiment makes us believe that such a cognitive strategy was not adopted. After all, that would require that the participants would be cognitively aware of the exact decline in maximum jumping distance as a result of the squatting. Hence, we tentatively concluded that the participants’ perception was adequately adjusted to the temporary decline in their physical capacities.

If we for the sake of argument assume this, then it would raise the question of how affordance perception and the perception of the environment in metric units are related. Although affordances might be the primary objects of perception, we can of course also perceive the environment in terms of meters and angles. However, the fact that affordance perception stays adaptive after an intense physical exercise while the accuracy of the perception of the steepness of a hill is often affected by it, suggests that the two perceptual judgments are independent (see also, Norman, 2002; Thomas et al., 2017; Withagen & Chemero, 2012)—the perception of the physical dimensions of the world is not based on the perception of affordances, and vice versa. However, because the studies on fatigue and perception examined either the perception of the physical dimensions of the world *or* the perception of affordances, they do not equip us to draw a strong conclusion on this. A critical test of this hypothesis awaits an experiment in which the participants have to judge both aspects (e.g., distance in meters *and* maximum jumping distance) and test whether and how each of them is affected by fatigue or any other change of the action system.

### The accuracy of the perceptual judgments

The accuracy of the perceptual judgments of the maximum jumping distance that we observed in our study is often higher than the ones that are reported in earlier studies. The studies by Cole et al. (2013) and Day et al. (2015) found that before performing a leaping action, adults underestimated their leaping distance; however, after practice performing the leaping action, participants perceptual judgments became more accurate. In contrast, Wagman et al. (2016) found people overestimating their leaping capacity when they have unlimited time to make the judgment, but observed that they become quite accurate when they have to give their judgments within 2 seconds (see also Heft, 1993, for a similar finding in the paradigm of reaching distance). In addition, Wagman et al. also demonstrated that the perception of maximum leaping distance was accurate when the leaping task was embedded in a “connects-the dots” puzzle task.

At present, it is unclear what explains the differences in accuracies found between the experiments. In all of these studies, contrary to ours, participants had to perform a leap (one-footed jump), with the preferred leg leading. However, the methods that were used to determine the perception of the maximum leaping distance differed. In Day et al.’s (2015) study, for example, participants had a remote control by which they themselves could move a little car to the maximum distance jumpable. In the study by Wagman et al. (2016), on the other hand, a dowel was placed at a certain distance from the participant, and he or she had to judge whether that distance was jumpable.

The present study differed from the above-mentioned experiments in that we tested a two-footed jump rather than a one-footed leap. Moreover, contrary to Wagman et al.’s (2016) study, we did not move the dowel in a step-wise way, asking the participant at each distance whether the maximum distance jumpable was reached; rather, the dowel was moved in a continuous way towards or away from the participant. Both the type of jump used and the way the perception of the maximum jumping distance was measured might be factors that could explain the differences in accuracy that were observed between our study and the above mentioned. Although we do not have a priori reasons to believe that people are better in estimating the maximum jumping distance in a two-footed jump than in a one-footed leap, it is also not a factor that we can exclude at this stage. However, we consider the method that we used a more likely candidate for explaining the high average accuracy of the affordance perception in our study. In line with the timed conditions of Wagman et al. (2016) and Heft (1993), participants had to be quick in judging whether the indicated distance was jumpable. After all, in our study, the dowel was moving continuously towards or away from them. Hence, the time constraint that we imposed in the present experiment might have prevented the participants from making an “analytic, reflective judgment” (Heft, 1993, p. 255). And, as both the study of Wagman et al. (2016) and Heft (1993) revealed, this contributes to the accuracy of the affordance perception.

### Concluding remarks

In line with an earlier study on (the perception of) the maximum reaching distance (Pijpers et al., 2007), we observed that on average participants are capable of accurately perceiving how far they can jump after an intense physical exercise. Also in the recovery of this exercise, the perception of the distance jumpable closely followed the regaining action potentiality. This finding is in keeping with the evolutionarily inspired approach of Gibson, which holds that animals adaptively perceive the affordances in their environments. The accuracy of the perception of steepness in angles and distance in meters might be affected by changes in the action capabilities of the participants (e.g., Bhalla & Proffitt, 1999; Proffitt et al., 2003); however, here we show that the accuracy of the perception of the maximum distance jumpable is not.

## Footnotes

1 To test whether perceived and actual maximum jumping distance differed across the sessions a 2 (perception vs. action session) × 6 (time) ANOVA was performed. A repeated-measures ANOVA showed no main effect of session on maximum jumping distance, *F*(1, 35) = 0.33, *p* = .568, and no Session × Time interaction, *F*(3.11, 108.91) = 0.26, *p* = .860. This confirms that participant’s maximum jumping distance did not differ between perception and action sessions. As expected, there was a significant main effect of time, *F*(1.70, 59.42) = 64.67, *p* < .000, η_p_^2^ = .65, η_G_^2^ = .28.
